# The outcomes of isolated ureteral resection and reconstruction in non-urologic cancer patients who underwent cytoreductive surgery (CRC) and hyperthermic intraperitoneal chemotherapy (HIPEC)

**DOI:** 10.1186/s12957-019-1770-x

**Published:** 2019-12-26

**Authors:** Şevket Barış Morkavuk, Murat Güner, Mesut Tez, Ali Ekrem Ünal

**Affiliations:** 1 Department of Surgical Oncology, Ankara City Hospital, Üniversiteler Mahallesi, Bilkent Caddesi N0 = 1, Onkoloji Binası, 6.Kat, Ankara, Turkey; 2 Department of General Surgery, Usak Research and Training Hospital, Usak, Turkey; 3 Department of Surgical Oncology, Ankara University Faculty of Medicine, Ankara, Turkey

**Keywords:** Ureteral resection, Cytoreductive surgery, HIPEC

## Abstract

**Background:**

Urinary system resections are performed during the cytoreductive surgery with hypertermic intraperitoneal chemotherapy (CRS-HIPEC). However, isolated ureter resection and reconstruction results are uncertain. The aim of this study was to evaluate the postoperative outcomes of isolated ureteral resection and reconstructions in patients who underwent CRC and HIPEC procedure.

**Methods:**

A total of 257 patients that underwent CRC and HIPEC between 2015 and 2017 in the Department of Surgical Oncology, Faculty of Medicine, Ankara University, were retrospectively analyzed. Twenty patients that had undergone isolated ureteral resection and reconstruction were included in the study. Predisposing factors were investigated in patients who developed postoperative complications.

**Results:**

The mean age of the patients was 55.1 years. The mean follow-up time of all the patients was 11.6 months. Postoperative mortality occurred in two patients. The mean PCI score was 13.9. Postoperative urologic complications were observed in eight patients after ureter reconstruction. There was no statistically significant difference between the groups in terms of reconstruction techniques and postoperative complications (*P* = 302). There was no correlation between age (*P* = 0.571) and gender (*P* = 0.161) with complications. CRS-HIPEC was performed mostly due to gynecologic malignancy. However, there was no correlation between the primary cancer diagnosis and the development of complications (*P* = 0.514). The hospital stay duration was higher in the group with complications (16.3 vs 8.8 days, *P* = 0.208).

**Conclusions:**

Ureteral resections and reconstructions can be performed for R0/1 resections in CRS-HIPEC operations. It leads to an increase in hospital stay. But there is no significant difference in the development of complications. In the management of complications, conservative approach was sufficient.

## Background

Peritoneal carcinomatosis is a disease characterized by peritoneal surface involvement of cancer cells. It is particularly seen in the late stages of gastrointestinal, gynecological cancers, and primary peritoneal malignancies. In selected patients, cytoreductive surgery (CRC) and hyperthermic intraperitoneal chemotherapy (HIPEC) are used for definitive treatment [1]. Respectively, due to the long operating time, multiple organ resections and chemotherapeutic agents, the CRC-HIPEC treatment has high morbidity (14–56%) and mortality (0.8–5%) rates [2]. Urinary system resections (bladder resection, nephrectomy, prostatectomy, and ureteral resection) are necessary at a rate of 7–20% during the CRC-HIPEC procedure [3]. However, in the literature, there is no sufficient data about long-term outcomes of these urological interventions. A few studies reported that these interventions have increased postoperative morbidity, mortality, and hospital stay rates, but did not change overall survival rates [4]. On the other hand, there is no data in the literature about postoperative course of isolated ureter resection and reconstruction.

The aim of this study was to evaluate the postoperative outcomes of isolated ureteral resection and reconstructions in patients with non-urologic peritoneal cancer who underwent CRC and HIPEC procedure.

## Material and method

### Patients

A total of 257 patients that underwent CRC and HIPEC between 2015 and 2017 in the Department of Surgical Oncology, Faculty of Medicine, Ankara University, were retrospectively analyzed. Twenty-six patients were found who had undergone diversion or reconstruction procedures due to urinary system involvement. Two patients with peritoneal cancer index (PCI) above 20 and four patients that had undergone nephrectomy were excluded from the study. Twenty patients that had undergone isolated ureteral resection and reconstruction were included in the study.

### CRC and HIPEC procedure

CRC procedure were performed as described by Sugarbaker [5]. Anastomoses were performed subsequent to HIPEC procedure. After placing an abdominal catheter, abdomen was closed and HIPEC was applied. Cisplatin (90 mg/m^2^) and mitomycin-C (15 mg/m^2^) were administered to the patients with ovarian cancer using closed abdomen technique at 42° for 60 min. For the patients with other cancer types, a combination of mitomycin-C (15 mg/m^2^) and carboplatin (300 mg/m^2^) was applied at 42° for approximately 60 min.

### Ureteral reconstruction

Ureteral reconstruction was performed with ureteroureterostomy, transureteroureterostomy, ureteroneocystostomy, and Boari flap methods. Ureterosigmoid anastomosis was performed in patients that underwent pelvic exenteration. In this method, loop colostomy had been created in the first step; then, distal 5 cm of sigmoid loop colostomy was used as a conduit. Both ureters were anastomosed to the loop colostomy with a double-j catheter by using the Bricker technique. In this way, a second ostomy was not required, and a second intestinal loop was not used as a conduit. In addition, fecal contamination was prevented because the ureteric anastomosis was performed distally to the ostomy.

### Data analysis

All statistical analyses were performed with SPSS v22.0. Mann-Whitney *U* test was used for comparing continuous data. Fisher’s exact test was used to compare categorical variables. Statistical significance was defined as *P* < 0.05.

## Results

### Patient profile

In this study, 20 patients that underwent isolated ureter resection and reconstruction in CRC and HIPEC procedures were evaluated. When the demographic data were examined, the mean age of the patients was 55.1 years (range 32–87). Fourteen patients (70%) were females, and six were males (30%). The mean age was 54.2 years (range 37–78) for women and 57.3 years for men (range 32–87). Concerning the primary cancer diagnoses, 10 patients had gynecologic malignancies, seven had colorectal cancer, and three had retroperitoneal sarcomas. Fourteen patients (70%) had undergone laparotomy in a different center and were referred to our clinic for CRC and HIPEC. Using preoperative imaging methods, hydronephrosis was detected in 12 patients. Hydronephrosis was on the left for five patients, right for four patients, and bilateral in seven patients (Table 1).

**Table 1:** Demographic distrubition of the patients

Sex	*n*(%)
Male	6 (%30)
Female	14 (%70)
Age, year, mean ± SD	55.15 ± 14 (32–87)
Primary cancer diagnosis	
Colorectal cancer	7 (%35)
Gynecologic cancer	10 (%50)
Retroperitoneal sarcoma	3 (%15)
Preoperative hydronephrosis	
No	8 (%40)
Right	4 (%20)
Left	5 (%25)
Bilateral	3 (%15)
Visceral organ resection	
Low anterior resection	7 (%35)
Total proctocolectomy	2 (%10)
Total colectomy	1 (%5)
Hemicolectomy	2 (%10)
A.P.R	2 (%10)
Pelvic exenteration	3 (%15)
TAH + BSO	3 (%15)
Types of ureteral reconstructions	
Ureteroureterostomy	2 (%10)
Trans ureteroureterostomy	6 (%30)
Ureteroneocystostomy	5 (%25)
Boari flap	4 (%20)
Ureterosigmoidostomy	3 (%15)
PCI, mean ± SD (range)	13.9 ± 3 (10–20)

*SD* standard deviation, *PCI* peritoneal carcinomatozis index, *Min* minimum, *Max* maximum, *A.P.R* abdomino perineal resection, *TAH + BSO* total abdominal hysterectomy + bilateral salpingo-oophorectomy

The mean follow-up time of all patients was 11.6 months (2 weeks–24 months) (Table 2). In hospital mortality occurred in two patients (2 of 20 patients). Eighteen patients were discharged and 5 of 18 patients died during follow-up period (mean survival time 11.6 months). Thirteen patients are still alive. Mean follow-up period for these 13 patients is 13.3 months.

**Table 2:** Follow-up time and length of hospital stay of all patients

Follow-up time, month, mean ± SD (range)	11.65 ± 8 (1–24)
Lenght of hospital stay, day, mean ± SD (range)	11.9 ± 8 (6–32)

### Surgical and postoperative outcomes

Multiple organ resection was performed in 20 patients. The mean PCI score was 15.2 (range 12–20) for the patients with ovarian cancer and 12.6 (range 10–15) for those with colorectal cancer and retroperitoneal sarcoma. The median completeness of cytoreduction (CCR) index was 0.

When the urological surgical procedures were examined, it was determined that left ureteral resection had been performed in seven patients (35%), right ureteral resection in eight patients (40%), and bilateral partial ureteral resection in five patients (25%). The reconstruction methods applied were ureteroureterostomy, transureteroureterostomy, ureteroneocystostomy, Boari flap procedure, and ureterosigmoidostomy.

The mean length of hospitalization was 11.9 days (range 6–32). Early mortality was seen in two patients (10%), of whom one died due to pulmonary thromboembolism on the 15th postoperative day and the other on the 25th postoperative day due to disseminated intravascular coagulation.

According to Clavien-Dindo classification, major complication was seen in seven patients. Early mortality was seen in two patients (grade 5), and anastomosis leakage was detected in five patients (grade 3a). There were no complications seen due to intestinal anastomosis.

Postoperative urologic complications were observed in eight patients after ureter reconstruction. Early anastomosis leakage was detected in five patients (10%) and late anastomosis stricture in three patients (15%).

There was no statistically significant difference between the groups in terms of reconstruction techniques and postoperative complications (*P* = 0.302). In the anastomotic leakage group, there were two patients with Boari flap (40%), one patient with ureteroureterostomy (20%), one patient with transureteroureterostomy (20%), and one patient with ureteroneocystostomy (20%). Three patients that developed late stricture had undergone transureteroureterostomy (Table 3).

**Table 3:** Factor affecting urological complications

	Urological complications (+)	Urological complications (−)	Total	*P* value
Age (years)	52.6	56.8	55.1	0.571
Sex	8	12	20	0.161
Male	4	2	6	
Female	4	10	14	
Primary cancer diagnosis	8	12	20	0.514
Colorectal c.	3	4	7	
Gynecologic c.	3	7	10	
Retroperitoneal	2	1	3	
Primary/recurrence	8	12	20	0.642
Primary	3	3	6	
Recurrence	5	9	14	
Hydronephrosis	8	12	20	0.126
No	3	5	8	
Yes	5	7	12	
Ureteral reconstructions	8	12	20	0.302
Ureteroureterostomy	1	1	2	
TransUreteroureterostomy	4	2	6	
Ureteroneocystostomy	1	4	5	
Boari flap	2	2	4	
Ureterosigmoidostomy	0	3	3	
PCI	13.5	14.1	13.9	0.521
Colorectal c.	13.3	13.2		
Gynecologic c.	15.3	15.1		
Retroperitoneal s	11	11		
Length of hospital stay (day)	16.3	8.8	11.9	0.208

Of the eight patients with postoperative complications, four had transureteroureterostomy (50%), two Boari flaps (25%), one ureteroureterostomy (12.5%), and one ureteroneocystostomy (12.5%).

Concerning hospital stay duration, there was no statistically significant difference between patients who developed complication and who did not (*P* = 0.208). The mean hospital stay was 16.37 ± 10 days (range 7–32) in the group with complications and 8.83 ± 2 days (range 6–13) in the group without complications.

The mean PCI was 13.5 in the complication group and 14.16 in the non-complication group, and there was no statistically significant difference between the two groups (*P* = 0.571).

For the management of complications, a unilateral percutaneous nephrostomy catheter was placed in patients who had anastomotic leakage. Bilateral percutaneous nephrostomy catheters were applied to patients that had undergone transureteroureterostomy. On a daily basis, urea creatinine was analyzed and compared both from abdominal drainage and the nephrostomy catheter. The anastomotic leakage was controlled by percutaneous nephrostomy in all patients. One month later, the nephrostomy catheters were withdrawn from the patients after confirming anastomosis integrity by opaque imaging methods.

In the evaluation of patients with late complications, hydronephrosis was observed in one patient at the fourth month, one patient at the fifth month, and one patient at the sixth month. These patients were first inspected using aureteroscope with a small diameter (< 8F). Balloon dilatation and double-j recatheterization were performed due to the stricture in the anastomosis line.

Mean survival was shown by Kaplan-Meier curve. There was no statistically significant difference between the groups about mean survival (log rank *P* = 0.710). Two-year survival ratio through a month is shown in (Table 4).

**Table 4:**
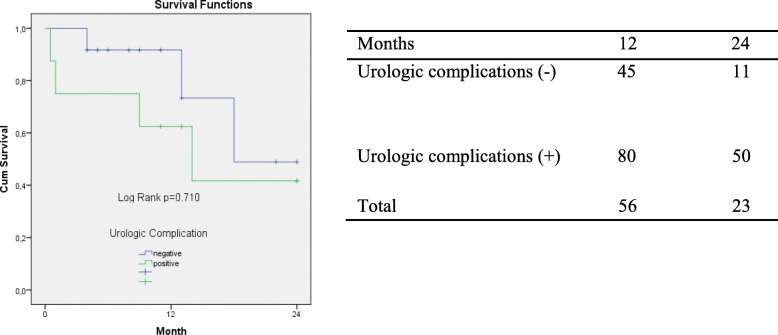
Overall survival according urological complications and average survival rates by months

## Discussion

CRC combined with HIPEC is an increasingly accepted curative-intent option for selected patients with peritoneal carcinomatosis. This combined procedure is associated with high rates of potentially life-threatening complications. It has a complication rate of 50–70%. Due to these high mortality and morbidity rates, the identification of patients who are more likely to benefit from CRS/HIPEC is of great clinical relevance. PCI and CCR are widely used in patient selection process, but these scores do not provide any information of the potential impact of an associated urologic procedure on both operative and long-term outcomes.

In our study, patients with a PCI value of 20 or below were included in the study group while those with a PCI greater than 20 underwent a debulking surgery to reduce tumor burden. Patients with a PCI value of 20 or higher are considered to have a poor prognosis. The common opinion is that this group of patients will not benefit from the CRC-HIPEC treatment [6]. There are studies on PCI and CCR in cases with various cancer diagnoses. Goere et al. reported the PCI cut-off value for survival as 17. In that study, patients that underwent palliative surgery using CRC-HIPEC were compared and it was found that this combined technique did not have any significant effect on the mean survival rate of patients with a PCI greater than 17. At the same time, authors also determine that postoperative complications were significantly higher in the CRC-HIPEC group [7].

Aggressive surgery to achieve CCR 0/1 increases the risk of morbidity and mortality in patients with high PCI values. In a study by Honere et al. found that the incidence of urinary fistula after urinary tract resection was significantly higher in patients with a PCI value above 20 [3]. To the best of our knowledge, there is no study in the literature investigating the PCI values after isolated ureteral reconstruction. In the current study, we did not find any relationship between PCI height and complication after ureteral reconstruction. Contrary to expectation, the mean PCI was higher in without complications group.

During the CRC procedure, urinary system resections are performed at a rate of 7–20% to achieve clean surgical margins or due to iatrogenic injury [3]. Similar to the literature, in our study, urinary system resection was performed in 10.1% (26 patients) of 257 patients who underwent CRC-HIPEC for the purpose of obtaining clean surgical margins. In addition, 7.7% of the patients (*n* = 20) underwent isolated ureter resection.

There are publications in the literature evaluating the length of hospital stay after urinary system interventions with CRC-HIPEC. However, we did not find any studies on the effect of isolated ureteral reconstruction on hospital stay. In a study by Lyon et al., the duration of hospitalization was significantly longer in patients that underwent a urological procedure (cystectomy, nephrectomy, ureteral repair, ureteral reconstruction, seminal gland resection) [8]. In another study conducted by Tan et al., hospital stay was found to be longer in patients that underwent a urological intervention [9]. Nevertheless, in both studies, the patients were grouped according to urologic interventions, rather than complications. The complication development and length of hospital stay were not correlated. In contrast, we grouped the patients according to the presence of complications after isolated ureter reconstruction. We found a longer hospital stay in the group with complications, albeit with no statistical significance.

In the evaluation of complications after ureteral reconstruction, early period anastomotic leakage was seen at a higher rate in patients that had received a Boari flap, and late period stricture in those that had undergone transureteroureterostomy. In transureteroureterostomy, the iatrogenic incision made in the ureter tissue means an additional anastomosis, which, we think, increases the development of strictures in the following period. Wenske et al., who compared the ureteroneocystostomy, Boari flap and psoas hitch flap methods, reported that minor and major complications were rare and there was no significant difference between these techniques in terms of the incidence of complications [10]. In another study, Iwaszko et al. observed that the most common complication after transureteroureterostomy was anastomotic leakage. A delayed anastomotic stricture developed in 4% of the cases, of whom 10% required relaparotomy during the 6-year follow-up [11].

Studies have shown that the possibility of postoperative complications is increased in CRC-HIPEC cases undergoing a urinary system intervention. Lyon et al. reported that the rate of complications was significantly higher following urinary system interventions compared to cases that did not undergo such an intervention [8]. Early complications include anastomotic leakage, obstruction, fistula, and pyelonephritis while obstruction and urinary tract stones are usually seen as late complications. In our study, only complications related to ureteral reconstruction were examined. In our patients, anastomotic leakage was observed as an early complication and stricture as a late complication. In the management of complications, the conservative approach was sufficient, and relaparotomy was not required. Anastomotic leakage was controlled by percutaneous nephrostomy. For the management of strictures, after ureteroscopy, ureteral continuity was maintained through balloon dilatation. In the literature, Leapman et al. treated ureteral obstruction and stricture using double-j catheterization, and Honore et al. used percutaneous nephrostomy for the treatment of ureteral fistulas [3, 4].

We did not find any relationship between hydronephrosis and complications after ureteral reconstruction. Guang et al. showed that the presence of preoperative hydronephrosis was not a factor in the development of urologic complications [12]. Similarly, in a study by Leapmanet al., three of 30 patients with postoperative urologic complications had preoperative hydronephrosis [4]. In our study, we observed that the presence of hydronephrosis or lateralization did not increase the development of complications after ureteral reconstruction.

In our cases of ureterosigmoidostomy anastomoses, we preferred to use the Bricker technique. The Bricker and Wallace methods are frequently used for ureterointestinal anastomoses. In a large-scale meta-analysis of 658 patients with a total of 1217 anastomoses, the Bricker and Wallace techniques were compared and did not significantly differ in terms of the development of complications [13]. In the current study, early and late complications were not observed in any of the patients that had undergone ureterosigmoidostomy.

Our study has certain deficiencies and lack of data. Due to its retrospective nature, some patients could not be included in the study. Therefore, a relatively small number of patients were examined. Furthermore, our 5 years survival rates have not yet been clarified since the patient follow-up continues.

## Conclusion

In cases with appropriate PCI and CCS values, ureteral resection and reconstruction can be safely performed during CRC-HIPEC. Despite the increase in hospitalization duration, there is no significant increase in complication development. Various parameters have been studied regarding postoperative complications but we did not identify any predisposing factor. There is also no difference between reconstruction methods in terms of complications. Possible complications can be controlled by the conservative approach or relaparotomy.

## Data Availability

The datasets used and/or analyzed during the current study are available from the corresponding author on reasonable request.
